# Members of the TEAD family of transcription factors regulate the expression of *Myf5* in ventral somitic compartments

**DOI:** 10.1016/j.ydbio.2011.04.005

**Published:** 2011-07-15

**Authors:** Ricardo Ribas, Natalia Moncaut, Christine Siligan, Kevin Taylor, Joe W. Cross, Peter W.J. Rigby, Jaime J. Carvajal

**Affiliations:** Section of Gene Function and Regulation, The Institute of Cancer Research, Chester Beatty Laboratories, 237 Fulham Road, London, SW3 6JB, UK

**Keywords:** *Myf5*, *Mrf4*, TEAD, Myogenic regulatory factor, Myogenesis, Somite, Dermomyotome, Transcription regulation

## Abstract

The transcriptional regulation of the *Mrf4/Myf5* locus depends on a multitude of enhancers that, in equilibria with transcription balancing sequences and the promoters, regulate the expression of the two genes throughout embryonic development and in the adult. Transcription in a particular set of muscle progenitors can be driven by the combined outputs of several enhancers that are not able to recapitulate the entire expression pattern in isolation, or by the action of a single enhancer the activity of which in isolation is equivalent to that within the context of the locus. We identified a new enhancer element of this second class, ECR111, which is highly conserved in all vertebrate species and is necessary and sufficient to drive *Myf5* expression in ventro-caudal and ventro-rostral somitic compartments in the mouse embryo. EMSA analyses and data obtained from binding-site mutations in transgenic embryos show that a binding site for a TEA Domain (TEAD) transcription factor is essential for the function of this new enhancer, while ChIP assays show that at least two members of the family of transcription factors bind to it *in vivo*.

## Highlights

► The ECR111 enhancer controls *Myf5* expression in ventral somitic compartments in early development. ► A TEAD binding site is essential for the activity of the ECR111 enhancer. ► ChIP analyses show that TEAD factors bind *in vivo* to the ECR111 enhancer. ► TEAD proteins localise to the myotome and the dermomyotome during early development.

## Introduction

The four myogenic regulatory factors (MRFs), Myf5, Mrf4, MyoD and Myogenin, members of the basic helix–loop–helix (bHLH) superfamily of transcription factors, play a key role in vertebrate skeletal myogenesis. In the mouse *Myf5* is the first MRF to be expressed and it is considered to be a skeletal muscle determination gene. It has been shown that in some muscle precursors Mrf4 and MyoD also behave as determination factors (Kablar et al., 1999; Kablar et al., 2000; Kassar-Duchossoy et al., 2004; Tajbakhsh et al., 1996) and they, together with Myf5, have chromatin remodelling properties (Bergstrom and Tapscott, 2001; Gerber et al., 1997).

In the mouse, *Myf5* is first detected around 8.0 days *post-coitum* (d*pc*) in the dermomyotomal dorso-medial lip and by 9.5 d*pc* it appears in the ventro-lateral lip, the myotome, and the branchial arches (Ott et al., 1991; Summerbell et al., 2000). By 10.5 d*pc Myf5* is also expressed in the developing limbs and the myoblasts migrating through the hypoglossal cord. Its expression is then maintained in all skeletal muscle precursors until it is down-regulated at late gestation stages. In the adult, *Myf5* is only expressed in satellite cells and muscle spindles (Zammit et al., 2004). *Mrf4* expression is biphasic. The embryonic phase starts at 9.0 d*pc* with expression detected in differentiated myocytes located in the central portion of the myotome of rostral somites. By 9.5 d*pc* (24–28 somites), *Mrf4* expression is activated in undifferentiated cells of the ventral somitic compartment, coinciding with or closely preceding *Myf5* expression at this location (Summerbell et al., 2002). By 10.5 d*pc*, *Mrf4* expression extends throughout the myotome but is absent from muscle progenitors of the branchial arches and the limbs. *Mrf4* expression is then downregulated in a rostrocaudal direction before the onset of the foetal phase of expression which is initiated in the limbs, followed by an upregulation of *Mrf4* expression in all skeletal muscles (Carvajal et al., 2001).

The genetic networks that control myogenesis have been shown to vary depending on the mesoderm of origin of the particular progenitor. Thus myogenesis of trunk precursors, that originate from segmented mesoderm, requires Pax3 (Tajbakhsh et al., 1997; Bajard et al., 2006; Brunelli et al., 2007) and is induced by Wnt signalling (Anakwe et al., 2003; Abu-Elmagd et al., 2010; Galli et al., 2004; Geetha-Loganathan et al., 2005; Munsterberg et al., 1995), while the expansion of muscle progenitors in the myotome has been shown to be modulated by Notch and Myostatin signalling interfering with terminal differentiation (Manceau et al., 2008; Schuster-Gossler et al., 2007; Vasyutina et al., 2007). Myogenesis in facial precursors that originate from migratory mesodermal populations that transit the branchial arches requires Tbx1 (Sambasivan et al., 2009) and is inhibited by Wnt signalling (Tzahor et al., 2003). Myogenesis in facial muscle precursors that give rise to extraocular muscles, which originate from unsegmented pre-chordal mesoderm, requires Pitx2 (Dastjerdi et al., 2007; Dong et al., 2006; Shih et al., 2007) while other facial muscles of splachnic mesodermal origin require Isl-1 (Nathan et al., 2008).

The requirement for a particular signalling environment or transcription factor in myogenesis is thus linked to the activation of survival or proliferative gene networks in progenitor cells and/or the activation of the MRFs which trigger differentiation. In this paper we show that a TEA Domain (TEAD) binding site is required for the activation of *Myf5* expression in a very precise subdomain within the ventral somite, further subdividing the already complex signalling environment of trunk muscle precursors. Finally we also show that at least two members of the TEAD family of transcription factors are bound *in vivo* to the enhancer, our interpretation being that this binding occurs through the essential TEAD binding site.

## Materials and methods

### Comparative analysis of genomic sequences

The genomic sequences for nine species (about 300 kb upstream and 50 kb downstream of the *Myf5* promoter) were analysed using the mVISTA bioinformatic tool (http://genome.lbl.gov/vista) and aligned with MLAGAN. The conservation parameters were set as follows [common name; *species* (% of conservation identity; minimum conservation window)]: human, *Homo sapiens* (70%, 100 bp); rhesus monkey, *Macaca mulata* (70%, 100 bp); cow, *Bos taurus* (70%, 100 bp); opossum, *Monodelphis domestica* (60%, 100 bp); chicken, *Gallus gallus* (55%, 100 bp); frog, *Xenopus tropicalis* (50%, 100 bp); medaka, *Oryzias latipes* (45%, 100 bp); zebrafish, *Danio rerio* (45%, 100 bp).

### Preparation of plasmid constructs

ECR111 was amplified by PCR from total mouse genomic DNA using the oligonucleotides: ECR111-F (5′ TTC AGG CTT GGG GGG AAG TC 3′) and ECR111-R (5′ CAA AAT AAC CAT TAG GAA TGC 3′). The Brain Enhancer (BE), as described by Daubas et al. (2009), was amplified using the oligonucleotides BE-F (5′ TCT AAG ATG AAC AGC AGC CTG A 3′) and BE-R (5′ TGG CTA CTC ATG AGA CTA TTT CA 3′). All PCR fragments were subcloned into the pCR2.1-TOPO vector (Invitrogen) and sequenced. We used a modified version of plasmid pEEBZ (Teboul et al., 2002) containing only the *Myf5* minimal promoter (Summerbell et al., 2000), a *nlacZ* reporter gene and a single cloning site upstream, to subclone the different enhancer elements. Full information on all cloning steps can be obtained on request.

### Homologous recombination in *Escherichia coli*

We generated a deletion cassette using the following oligonucleotides for the 5′ homology arm: 5HA-F, 5′ TCT AAA TTA TAT TGC ACA GAG TTC 3′ and 5HA-R, 5′ GAA CCA CCT TAA GAA CCA ATA C 3′; for the 3′ homology arm: 3HA-F 5′ tgg ttc tta agg tgg ttc TTT TCT TTT TAT GTG ACT ACA GTA A 3′ and 3HA-R, 5′ ATA TAA AAT GTG GAA AGA TCA GTC 3′. The lowercase in the 3HA-F oligonucleotide is complementary to the 5HA-R. After amplification individual homology arms were joined by PCR to generate the deletion cassette.

The cassette was then used to delete ECR111 from the BAC construct B195APZ (Carvajal et al., 2001) to generate B195APZ^∆111^ by linear recombination as previously described (Carvajal et al., 2008).

### Production of transgenic mice and whole-mount histochemistry

All *in vivo* experimentation was performed according to United Kingdom Home Office Regulations. BAC DNA was prepared using the QIAgen Maxiprep Kit (Qiagen) following our own modification of the protocol (Carvajal et al., 2001) and used to inject fertilised mouse eggs from CBA/Ca × C57Bl/6 crosses, as previously described (Yee and Rigby, 1993). Embryos were sectioned using a vibrotome at 70 μm following agarose embedding or using a cryostat at 10 μm following LAMB/OCT (Thermo Fisher Scientific) embedding following manufacturer's instructions. The protocols for ß-Galactosidase and Alkaline Phosphatase staining of mouse embryos have also been described elsewhere (Carvajal et al., 2001).

### Immunohistochemistry

For whole-mount immunohistochemistry, embryos were dehydrated through a methanol series (50% to 100% MeOH), fixed overnight in a 4:1 solution of MeOH and DMSO, incubated in a 4:1:1 solution of MeOH, DMSO, 30% (v/v) H_2_O_2_ for 4 h and rehydrated through a MeOH/PBT [PBS + 0.5% (v/v) triton X-100] series (50% to 15% MeOH). For immunostaining, embryos were incubated in PBTBG [PBT, 2% (v/w) BSA, 10% (v/v) goat serum] for 4 h. Individual TEAD antibodies (see below) were added (1:100–1:250 dilutions), embryos were incubated overnight at 4 °C, washed in PBT overnight at 4 °C and equilibrated in PBTBG. Fluorescent conjugated antibody (Alexa Fluor 488 goat anti-mouse IgG; 1:1000) was added and embryos were incubated overnight at 4 °C. Prior to visualisation, embryos were washed in PBT and transferred to 50% (v/v) glycerol.

For immunohistochemistry on sections, embryos were fixed overnight in 4% (w/v) PFA, embedded and sectioned. Sections were washed in PBT and incubated in PBTBG for 1 h. Individual TEAD antibodies (see below) were added (1:100–1:500 dilutions), incubated for 1 h at room temperature, followed by an overnight incubation at 4 °C and equilibrated in PBTBG. Fluorescent conjugated antibody (Alexa Fluor 488 goat anti-mouse IgG; 1:1000 or Alexa Fluor 488 goat anti-rabbit IgG; 1:1000) was added and sections incubated for 1 h at room temperature. Prior to visualisation, sections were washed in PBT.

### Electrophoretic mobility shift assays (EMSAs) and supershifts

EMSA experiments were performed as previously described (Gilthorpe et al., 2002). Whole-cell protein extracts were prepared from isolated trunks of 10.5 d*pc* mouse embryos. Specific competitors were added at 10- or 50-fold molar excess. For supershift experiments, anti-TEAD1 antibody was added to the reaction 15 min before the addition of the biotin-labelled probe. Full oligonucleotide sequences can be supplied upon request.

### Protein *in vitro* translation (IVT) and Western blotting

cDNAs for the four members of the TEAD family were cloned in the pcDNA3.1(−) vector and proteins synthesised *in vitro* using the TNT® Coupled Reticulocyte Lysate System (Promega) following manufacturer's instructions. At least two transcript variants exist for TEAD4 and we cloned the sequences equivalent to NCBI accession numbers NM_011567 (variant 1) and NM_001080979 (variant 2) and performed IVT on both together. IVT proteins were separated through a 10% SDS-PAGE gel, electro-blotted and blocked in 5% (w/v) dry milk. Primary and secondary antibody incubations were performed in 5% (w/v) dry milk. The antibodies used were as follows: mouse anti-TEAD1 (Aviva Systems Biology, ARP39521, 1: 2000; BD Biosciences, 610922, 1:1000); rabbit anti-TEAD2 (Santa Cruz Biotechnology, sc-67115, 1: 2000); rabbit anti-TEAD3 (Aviva Systems Biology, ARP33429, 1:1000); mouse anti-TEAD4 (Aviva Systems Biology, ARP38276, 1:2000; SantaCruz Biotechnology, sc-101184, 1:2000); goat anti-rabbit IgG (H + L)-HRP (BioRad; 1:2000) and goat anti-mouse IgG (H + L)-HRP (BioRad; 1:2000). Conjugated HRP was detected using the Amersham ECL plus Western Blotting Detection System (GE Healthcare).

### Chromatin immunoprecipitation (ChIP)

Trunks from 10.5 d*pc* embryos were cross-linked in 1% (v/v) formaldehyde at 4 °C for 20 min, the reaction was quenched with 125 mM glycine, and the embryos were homogenised and sonicated to give chromatin of 200–1000 bp. Chromatin was incubated overnight at 4 °C with a TEAD antibody or isotype controls (Mouse IgG: AbCam, Rabbit IgG: Santa Cruz Biotechnology) prebound to Protein G magnetic dynabeads (Invitrogen). Cross-linking was reversed by an overnight incubation in Elution Buffer [100 mM NaHCO_3_, 1% (w/v) SDS] at 65 °C, DNA purified using the QIAquick PCR purification kit (Qiagen) and amplified using the oligonucleotides: ChIP111-F: 5′ TTC AGG CTT GGG GGG AAG TC 3′, ChIP111-R: 5′ CAA AAT AAC CAT TAG GAA TGC 3′, ChIP-control-F: 5′ AAG TCC ACT ACC ATG GAT CGG 3′ and ChIP-control-R: 5′ GTC AGA GCA GTT GGA GGT GG 3′.

## Results

### Isolation of an enhancer that drives expression in ventro-caudal and ventro-rostral somitic compartments

**Fig. 1:**
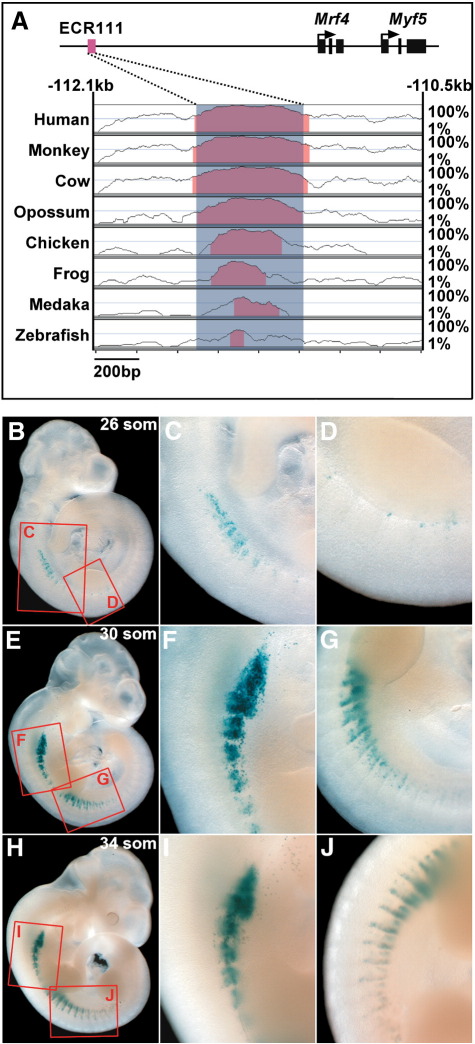
The ECR111 enhancer drives a highly dynamic somitic pattern. (A) Graphical representation of the location of ECR111 in relation to the *Mrf4* and *Myf5* genes and the output of the VISTA analysis of the − 112.1 kb to − 110.5 kb interval in the upstream region of the *Mrf4/Myf5* locus generated by comparing the mouse sequence (base genome) to that of 8 different vertebrate species. The red peaks represent conserved non-coding sequences that define the ECR111 enhancer. The position in relation to the transcription initiation site for *Myf5* is given (in kb) above the graph; numbers on the right indicate the percentage of conservation. (B, E, and H) Transgenic embryos carrying the ECR111-MZ construct and details of the cervical (C, F, and I) and thoracic (D, G, and J) aspects of the expression. (B−D) At 26 somite stage, the enhancer drives expression in the ventral half of cervical somites and at the base of limb-level somites. (E−G) At 30 somite stage there is an expansion of the domain of expression in cervical somites and an upregulation of transgene expression in thoracic somites; around half of interlimb somites express the transgene. Note the clear localisation of expression particularly at the caudal region, and the expression spanning the width of the somite at more ventral positions of cervical and thoracic somites. (H−J) By 34 somite stage, all thoracic somites express the transgene in caudal and rostral somitic regions, although expression extends more dorsally at the caudal domain. Red boxes in B, E and H indicate the close up regions shown in the remaining panels.

We have previously shown that the region located between − 140 kb and − 88 kb upstream of the *Myf5* transcriptional start site is required for the expression of both *Mrf4* and *Myf5* in ventral somitic compartments (Carvajal et al., 2001). Sequence conservation analysis using the VISTA bioinformatic tools (Dubchak and Ryaboy, 2006) identifies at least four evolutionarily conserved regions (ECRs) in this interval, one of which (ECR111, 527 bp, which corresponds to the E3 putative enhancer described by Maak et al., 2006), is located 111 kb upstream of *Myf5* and is conserved throughout vertebrate evolution being present in mammals, birds, amphibians and fish (Fig. 1A). As ECR111 is the most conserved sequence in the *Mrf4/Myf5* locus, we decided to concentrate first on the transgenic analysis of this putative enhancer. We cloned ECR111 upstream of the *Myf5* minimal promoter (Summerbell et al., 2000) and a *nLacZ* reporter gene (construct ECR111-MZ) and analysed the expression pattern driven by it in transgenic animals (n = 5, including 2 stable lines). At 9.25 d*pc* (23–24 somites), ECR111 drives expression in a few isolated cells in the cervical somites (Fig. S1A). The pattern driven by the transgene evolves rapidly and by 9.5 d*pc* (26 somites) expression is clearly observed in cervical somites and isolated cells at ventral positions in limb-level somites (Figs. 1B–D). By early 10.0 d*pc* (30 somites), there is a general upregulation of the cervical somitic expression and a rostro-caudal expansion of the thoracic domain, which follow the same pattern as the cervical somites and thus expression is mainly observed in the ventral half of somites predominantly in the caudal and rostral edges (Figs. 1E–G). By 10.5 d*pc* (34 somites) cervical somitic expression is maintained, or slightly reduced, while all thoracic somites express the transgene in rostral and caudal edges, including some expression in the ventral half of the myotome (Figs. 1H–J). From 11.5 d*pc* expression is downregulated so that by 12.5 d*pc* transgene activity can only be faintly detected in some shoulder and intercostal muscles (Fig. S1B) and is absent from 13.5 d*pc* onwards.

**Fig. 2:**
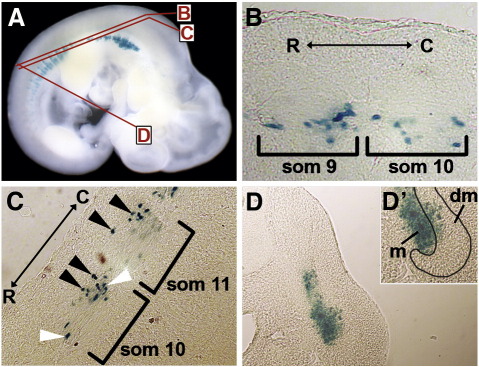
The ECR111 enhancer drives expression in the myotome and in the dermomyotome. (A) Transgenic embryo carrying the ECR111-MZ and transverse (B, C) or saggital (D) sections of the same embryo. (B) At more dorsal positions, expression is restricted to the myotome, particularly the caudal regions, while (C) at more ventral positions expression is observed in both the myotome (white arrowheads) and the dermomyotome (black arrowheads), again preferentially in the caudal somitic region. (D) Saggital section through an interlimb somite shows that the expression is mainly restricted to the myotomal compartment, with some cells expressing the transgene in the dermomyotome as shown in the close-up inset (D′). C: caudal; R: rostral; m: myotome; dm: dermomyotome. The position and orientation of the sections are shown as red lines in A. The somites are numbered according to their rostro-caudal position so that the most rostral is somite 1.

Within the somite, expression is observed in both the dermomyotomal and myotomal compartments, depending on the dorso-ventral level (Fig. 2). At more dorsal levels expression is restricted to myotomal myonuclei located in rostral and caudal positions, although nuclei located in the caudal part of the myotome are more prevalent (Fig. 2B). At more ventral somitic levels, expression is observed both in the myotome and in the dermomyotome (Fig. 2C, white and black arrowheads, respectively) in rostral and caudal positions. Once again, most of the nuclei expressing the transgene are located caudally within the somite, both in the myotome and in the dermomyotome. Transverse sections (Fig. 2D) confirm this observation, as the transgene drives expression in the ventral half of the myotome and only in the ventral quarter of the dermomyotome.

### ECR111 is necessary for *Myf5* ventral somitic expression, but not for *Mrf4* expression

**Fig. 3:**
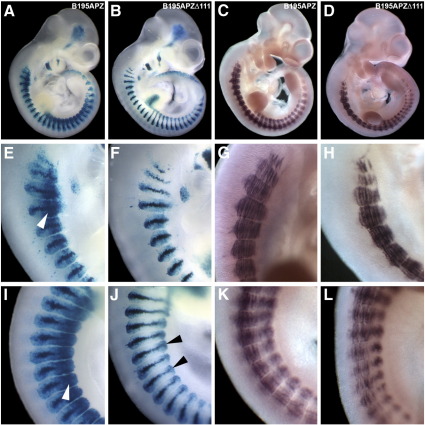
Deletion of ECR111 abolishes *Myf5* expression in caudal and rostral somitic regions. 10.5 d*pc* embryos from transgenic lines carrying the B195APZ wild type (A and C) or B195APZ^∆111^ (B and D) BAC constructs, and details of the expression patterns in cervical (E−H) and thoracic (I−L) somites. (B) Deletion of ECR111 from the BAC shows that this enhancer is necessary for *Myf5* expression in the caudal and rostral regions in cervical (F) and thoracic (J) somites, while expression in the somitic bud (J, arrowheads) and *Mrf4* expression are not changed in the absence of the enhancer (D, H, and L) when compared to the pattern driven by B195APZ (C, G, and K). Note the domain of expression that spans the ventral half of the somite in embryos carrying the wild type BAC construct (arrowheads in E and I).

Having shown that ECR111 is sufficient to drive expression in the ventral half of the somite, we deleted this sequence from BAC clone B195APZ, which we have previously shown to be able to drive the entire expression patterns of both *Mrf4* and *Myf5* in the embryo and in the adult (Carvajal et al., 2001; Zammit et al., 2004), and generated construct B195APZ^∆111^. This BAC contains *human placental alkaline phosphatase* (AP) and *nlacZ* (Z) as reporter genes for *Mrf4* and *Myf5*, respectively, and allows for the analysis of the effect of a single deletion on the expression of the two linked genes in transgenic mice (n = 2 stable lines). At around 9.5 d*pc* (24–26 somites), the transgene fails to drive *Myf5–nlacZ* expression in the ventral half of cervical and thoracic somites (data not shown), while there are no changes in the expression pattern of *Mrf4–AP* (Fig. S2A) compared to embryos carrying the wild type B195APZ BAC clone (Fig. S2B). By 10.5 d*pc*, *Myf5–nlacZ* expression in absent from the ventral half of the myotome in thoracic and cervical somites and the ventral quarter of the dermomyotome (Figs. 3B, F, and J, Fig. S2C), although clear expression can be observed in the ventral somitic bud (Fig. S2D), while *Mrf4–AP* expression is identical to that driven by the wild type construct (Fig. 3). Therefore, at these stages the expression pattern driven by ECR111 seems to be complementary to that driven by B195APZ^∆111^, and the sum of both patterns could result in the recapitulation of the entire expression pattern of *Myf5*. Interestingly, this does not occur at later stages (Fig. S2E and F) and thus neither the B195APZ^∆111^ or the ECR111-MZ transgenes drive *Myf5–nlacZ* expression in the ventral half of tail somites, indicating that this aspect of the expression, while being dependent on ECR111, cannot be driven by the enhancer in isolation.

### ECR111 contains a TEAD binding site necessary for enhancer activity

In order to refine the essential sequences for enhancer function, we proceeded to identify putative binding sites within ECR111 by electrophoretic mobility shift assays (EMSAs) using 34 bp overlapping oligonucleotides designed to span the most conserved regions within ECR111. Protein extracts were obtained from the trunk region of 10.5 d*pc* wild type (C57Bl6/CBA) embryos. Of the 26 double stranded oligonucleotides tested, 10 generated a distinct shift in this assay and we performed competition analyses using unlabelled oligonucleotides which show that these correspond to 6 independent binding sites (data not shown). These sites were named according to their position within the enhancer as BS-1 to BS-6.

The shift generated by oligo#2 (BS-1) was completely abolished by cold oligo#1 (data not shown) and thus the binding site was refined to the 17 bp interval defined by the overlap of these two oligonucleotides. Within this interval, there is a highly conserved canonical M-CAT binding site (Mar and Ordahl, 1990), known to be involved in the regulation of muscle-specific genes and recognised and bound by members of the TEAD family of transcription factors (Farrance et al., 1992). A cold oligonucleotide derived from the chicken *troponin-T* gene and containing a consensus binding site (Pasquet et al., 2006) efficiently abolishes the shift generated by oligo#2, while an oligonucleotide in which the binding site has been mutated (CATTCCT > ggTaCCT; oligo#2-m1) is unable to compete with wild type oligo#2, even at high molar excesses (Fig. S3A). Finally, a TEAD-1 antibody that binds to three members of the TEAD family (see below) recognises the protein (or protein complex) binding in our assay and generates a supershift (Fig. S3A). These data indicate that, at least in EMSA assays, a member of the TEAD family of transcription factors is able to bind to the 17 bp sequence defined by the overlap between oligo#1 and oligo#2. Oligo#25 (BS-6) also contains a M-CAT binding site but this was not efficiently competed with the chicken *troponin-T* oligonucleotide or oligo#25 mutated at the M-CAT binding site. Intriguingly, the band-shift was abolished by the anti-TEAD1 antibody (Fig. S3B). The remaining shifting oligonucleotides did not contain any obvious muscle-related binding sites and thus were further analysed by competition with overlapping oligonucleotides and with oligonucleotides mutated at three consecutive nucleotides in order to determine the sequence required for binding. In this way we predicted YY1 binding sites in oligo#7 (BS-2) and oligo#15 (BS-4), an AP-1/CBP/NF-Y binding site in oligo#9 (BS-3), and an AP-1/NF-E binding site in oligo#18 (BS-5). The location and sequences of the mutations, as well as the predicted protein binding sites, can be found in Fig. S4.

**Fig. 4:**
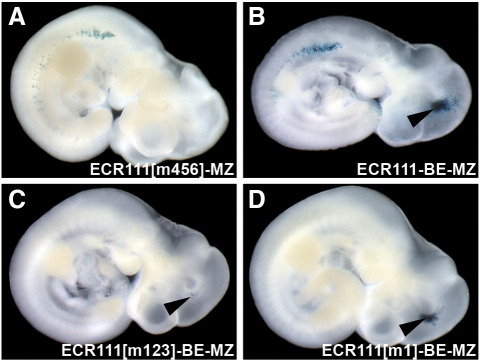
Transgenic mutation analysis shows that BS-1 is essential for enhancer function. (A) Mutation of the putative BS-4, BS-5 and BS-6 binding sites (construct ECR111[m456]-MZ) does not interfere with ECR111 activity at 10.5 d*pc*. (B) The ECR111-BE-MZ construct drives expression in the brain (arrowhead) and the somitic ECR111 domain. (C) Mutation of BS-1, BS-2 and BS-3 binding sites (construct ECR111[m123]-BE-MZ) or (D) the single mutation of BS-1, abolish ECR111-driven expression at 10.5 d*pc*, but not that driven by the brain element (BE) used as a transgenesis expression control (arrowheads in C and D).

We then introduced the mutations shown to abolish binding in EMSA assays into our ECR111-reporter plasmid construct in order to assess the requirement for these binding sites in the pattern driven by ECR111. The combined mutation of binding sites BS-4, BS-5 and BS-6 (construct ECR111[m456]-MZ) does not affect the expression pattern driven by ECR111 (Fig. 4A; n = 4), while we could not detect expression in any of the transgenic embryos carrying the construct ECR111[m123]-MZ, in which BS-1, BS-2 and BS-3 had been mutated (data not shown). In order to distinguish between lack of expression due to the introduced mutation or due to silencing at the integration site, we generated a new base construct which also carries the *Myf5* brain element (Daubas et al., 2009) as an internal control (construct ECR111-BE-MZ). This new construct is thus able to drive both somitic and brain expression (Fig. 4B). Mutation of BS-1 (M-CAT), BS-2 (YY1) and BS-3 (AP-1/CBP/NF-Y) (construct ECR111[m123]-BE-MZ) abolishes the function of ECR111 at 10.5 d*pc* as the construct is not able to drive somitic expression, while brain expression is maintained, indicating that the lack of expression is due to the mutations introduced (Fig. 4C; n = 3). Finally, mutation of BS-1 (M-CAT) in isolation (construct ECR111[m1]-BE-MZ) is sufficient to abolish ECR111 function, while not affecting the brain expression driven by the BE (Fig. 4D; n = 4). We found some variation in the levels of brain expression, which is normal in transient transgenic embryos, but ECR111-BE-MZ embryos that display very low levels of expression in the brain were always associated with somitic expression, while embryos carrying ECR111[m123]-BE-MZ or ECR111[m1]-BE-MZ never show somitic expression, even if high levels are detected in the brain. This shows that the M-CAT binding site is necessary for the function of the ECR111 enhancer.

### TEAD transcription factor family members bind to ECR111 *in vivo*

**Fig. 5:**
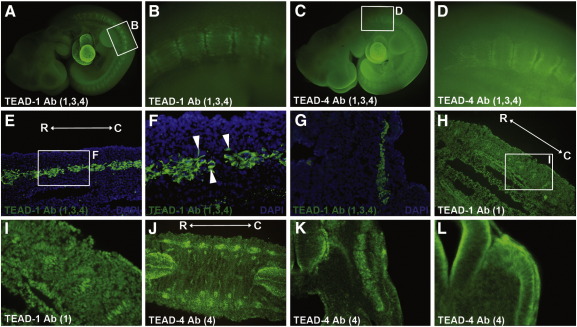
TEAD proteins localise to the myotome and the dermomyotome. Whole-mount immunohistochemistry with anti-TEAD1 (Aviva) (A and B) and anti-TEAD4 (Aviva) (C and D) shows that TEAD proteins in the 10.0 d*pc* embryo localise to the developing heart and the somites, where staining is stronger in caudal and rostral regions. Immunohistochemistry on sections from 10.5 d*pc* embryos with anti-TEAD1 (Aviva) (E–G), anti-TEAD1 (BD Biosciences) (H and I) and anti-TEAD4 (Santa Cruz) (J−L) shows that these TEAD proteins in the somite are preferentially localised to the myotome in interlimb regions, although some dermomyotomal cells at this level are also positive (F, arrowheads). At tail somite levels TEAD-4 seems to localise to the dermomyotome (L). (E, H, and J) are saggital sections at interlimb levels; (G and K) are transverse sections at interlimb levels; (L) is a transverse section at tail somite level. White boxes in A, C, E and H indicate the close up regions shown as B, D, F and I. The numbers in brackets refer to the members of the TEAD family recognised by the named antibody (see the text for information on the specificity of the TEAD antibodies).

We analysed the specificity of the different TEAD antibodies by western blot of *in vitro* translated proteins. TEAD-1 Ab (Aviva) is able to bind weakly to TEAD1, TEAD3 and TEAD4 (data not shown); TEAD-3 (Aviva) is able to recognise TEAD-1 and TEAD-3; TEAD-4 Ab (Aviva) recognised TEAD-1, TEAD-3 and TEAD-4; TEAD-2 Ab (Santa Cruz), TEAD-1 (BD Biosciences) and TEAD-4 (Santa Cruz) are highly specific for their respective TEAD factors (Fig. S5). We then performed whole-mount immunohistochemistry using the different antibodies against the TEAD family of transcription factors to determine where in the embryo the TEAD proteins are localised. The specific TEAD-2 Ab (Santa Cruz) did not show fluorescence in 10.0 d*pc* embryos, while TEAD-3 Ab (Aviva) generates very faint immunofluorescence in all somites (data not shown). In contrast, TEAD-1 and TEAD-4 antibodies (which recognise TEAD-1, 3 and 4) show clear protein localisation in the heart and the somites (Figs. 5A–D). Immunohistochemistry on sections of 10.5 *dp*c embryos shows that TEAD proteins mainly localise to the myotome, with isolated cells present in the dermomyotome (Figs. 5E and F). Interestingly the localisation of the protein is not restricted to the nucleus and strong cytoplasmic staining is observed (Fig. 5E and F). Using the specific TEAD-1 (BD Biosciences) antibody on sections shows that TEAD-1 in the somites is localised to nuclei from myotome and dermomyotome cells, mainly present at caudal regions (Figs. 5G–I). The specific TEAD-4 antibody (Santa Cruz) shows the presence of TEAD-4 throughout the myotome of somites at interlimb levels. In this case, staining is clearly observed in the cytoplasm of myotubes (Figs. 5I and J). Interestingly, transverse sections at interlimb level show that TEAD-4 is predominantly localised to the myotome (Fig. 5K), while in tail somites expression is restricted to the dermomyotome (Fig. 5L).

**Fig. 6:**
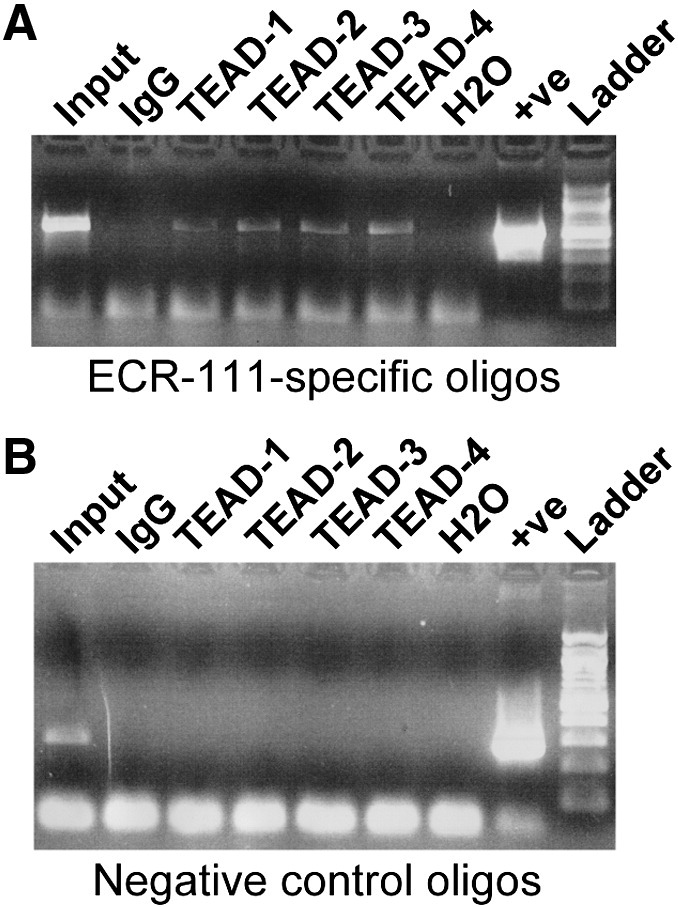
The ECR111 enhancer is bound *in vivo* by members of the TEAD family of transcription factors. ChIP analyses of (A) the ECR111 and (B) exon 1 regions show that all TEAD antibodies used are able to immunoprecipitate the ECR111 enhancer. See the text for information on the specificity of the TEAD antibodies. Input: sonicated chromatin prior to immunoprecipitation; H2O: no chromatin added to the reaction; + ve: total mouse genomic DNA; Ladder: 100 bp ladder (New England Biolabs).

We then performed ChIP assays on chromatin isolated from 10.5 d*pc* mouse embryo trunks to determine if a member of the TEAD family was binding to this enhancer *in vivo*. All TEAD-family antibodies were able to recognise proteins bound to the ECR111 region *in vivo* (Fig. 6A), while none were able to immunoprecipitate the DNA control region from exon1 of *Myf5* (Fig. 6B). ChIP experiments carried out with the more specific TEAD1 and TEAD4 antibodies failed to produce consistent results. As the antibodies that consistently immunoprecipitate ECR111 recognise more than one member of the family, we cannot determine at this stage exactly which TEAD members are binding *in vivo* to the enhancer, but combination of the western blot and ChIP data shows that: i) TEAD-2 is bound *in vivo* to the enhancer and ii) another TEAD member must also be binding to the enhancer.

## Discussion

We have previously shown that the transcriptional regulation of the *Mrf4/Myf5* locus depends on a series of enhancers that drive highly specific temporal and anatomical aspects of the expression of the two genes (Carvajal et al., 2001). The data clearly showed the existence of at least two classes of elements: enhancers that require the input of other enhancers in order to drive the correct expression pattern, and enhancers that are able to drive a particular aspect of the expression in isolation. ECR111 belongs to the latter group and constitutes the most evolutionarily conserved sequence within the locus. The results show that in isolation ECR111 controls a very specific aspect of the somitic expression of *Myf5*, the same aspect that is lost from the full expression pattern when the enhancer is deleted from the locus, and strikingly it unveils a further subdivision within the dermomyotomal lip domains. Finally, we show by EMSA, ChIP and transgenic analyses that a highly conserved TEAD binding site is essential for enhancer function and that members of the TEAD family of transcription factors are bound *in vivo* to the enhancer.

### The ECR111 enhancer controls ventro-caudal and ventro-rostral *Myf5* expression

We have previously shown that the − 140 kb to − 88 kb interval is required for the ventral somitic expression of *Myf5* and *Mrf4* (Carvajal et al., 2001). While in most skeletal muscle progenitors, *Myf5* expression precedes that of *Mrf4*, in the somitic bud (the most ventral aspect of the thoracic somites), the onset of *Myf5* expression coincides with or closely follows that of *Mrf4* (Summerbell et al., 2002). Because of this coincidence in time and space, we have previously hypothesised that a single enhancer may be responsible for the activation of both genes in the ventral somitic compartment (Carvajal et al., 2001), and we embarked on the characterisation of enhancers within this 52 kb interval. Comparative sequence analyses revealed the presence of at least four highly conserved sequences within the interval, one of which (ECR111) is conserved in all vertebrates, conservation that is higher than that of the coding exons of *Mrf4*, *Myf5* or the *Ptprq* gene*,* located immediately upstream and harbouring ECR111 in one of its introns. Transgenic animals carrying the ECR111-MZ construct express the reporter gene in the ventral half of the myotome and the ventral quarter of the dermomyotome of thoracic and cervical somites but not in the somitic bud, indicating that another enhancer must drive this aspect of the pattern. Furthermore, deletion of ECR111 from B195APZ (construct B195APZ^∆111^) confirms this finding as expression in the somitic bud is not affected and the only missing aspect of the expression coincides with that driven by the enhancer in isolation. Therefore, ECR111 belongs to the class of *Mrf4/Myf5* "modular enhancers" the function of which does not need additional input from other elements in the locus, placing it in the same class as the EEE element (Teboul et al., 2002), the brain element (Daubas et al., 2009) and the limb element (Hadchouel et al., 2003). While this is true for the thoracic somitic pattern, it is different in the tail somites where the enhancer in isolation is not able to drive ventral expression, nor is the construct B195APZ^∆111^, from which the enhancer has been deleted. The formation of tail somites is different from that of thoracic and cervical somites. In the mouse, the onset of tail somite formation occurs around 10.0 d*pc* (Wilson and Beddington, 1996) and ends around 13.5 d*pc*, when the total number of tail somites is reached (Tam, 1981). It has been shown that signals emanating from the ventral ectodermal ridge (Bmp2 and Fgf17), through the control of Wnt3a levels, are essential for somitogenesis and tail elongation (Goldman et al., 2000). Mice carrying the spontaneous mouse mutation *vestigial tail* (a hypomorphic Wnt3a mutation) or lacking Wnt3a (Greco et al., 1996) show reduced length or absent tail, respectively, while anterior somitogenesis is unaffected; it has also been shown that cervical and thoracic somites but not tail somites require cyclic *lunatic fringe* expression (Shifley et al., 2008; Stauber et al., 2009). Therefore the signalling requirements for tail somite formation are different from those of thoracic and cervical somite formation, while the basic process of somitogenesis is probably shared (reviewed in Pourquié, 2001). Our data indicate that the activation requirements for tail and thoracic somitic ventral *Myf5* expression are also different, as ECR111 is necessary but not sufficient to drive this aspect of the tail pattern, indicating that differences in somite formation may be reflected in the signalling requirements for myogenesis in the tail as compared to the thoracic and cervical somites.

### A TEAD binding site is essential for expression of *Myf5* in the ventral domain of caudal and rostral somitic edges

EMSA analyses showed several putative binding sites within ECR111 and by using competition analyses with overlapping and mutated oligonucleotides we refined the location of these sites. While binding-site prediction software is generally unreliable, due to the natural variation of these sequences and the very limited availability of fully characterised sites, our supershift analyses show that one of the sites identified by EMSA and predicted as a canonical M-CAT binding site, is bound in our *in vitro* assay by members of the TEAD family of transcription factors. Mutation analyses carried out in transgenic animals show that the TEAD binding site BS-6 is not required for enhancer function (construct ECR111[m456]-MZ), while embryos carrying the double enhancer construct ECR111[m1]-BE-MZ, in which the TEAD BS-1 site has been mutated to abolish binding, express the reporter gene in the brain, which acts as positive control but not in the somitic domains controlled by ECR111. This shows that the TEAD BS-1 site is absolutely required for ECR111 enhancer function at 10.5 d*pc*. Nevertheless, a 200 bp sub-fragment of ECR111 carrying this binding site and the immediately downstream BS-2 and BS-3 sites, was not able to drive transgene expression (Ribas and Carvajal, unpublished observations). As the construct in which the remaining sites identified by EMSA were mutated (ECR111[m456]-MZ) was able to drive the entire ECR111 pattern, this suggests that another site(s) not identified in our EMSA assay may also be required. Although it is known that in some cases TEAD proteins may bind in isolation, it has also been shown that their activity often depends on the binding of other factors or co-factors such as Srf, Mef2, vestigial-related proteins, bHLH proteins, TAZ (transcriptional co-activator with PDZ-binding motif), or YAP (Cao et al., 2008; Farrance and Ordahl, 1996; Gupta et al., 1997; Gupta et al., 2001; Larkin et al., 1996; Lian et al., 2010; Maeda et al., 2002a; Maeda et al., 2002b; Mahoney et al., 2005; Ota and Sasaki, 2008; Sawada et al., 2008; Zhao et al., 2008). It is possible to speculate that a second transcription factor, that forms a complex with the TEAD protein, is acting through the binding site(s) present within oligo#25, as these are supershifted by increasing concentrations of anti-TEAD1 antibody. It is also possible that the second TEAD binding site at this location is active, but with a higher affinity for the TEAD protein than the chicken *troponin-T* consensus sequence, although the partial competition by mutated oligo#25 will argue against this possibility.

By using chromatin immunoprecipitation (ChIP) assays we have also shown that members of the TEAD family of transcription factors bind ECR111 *in vivo*, and the obvious interpretation is that this binding occurs through the TEAD BS-1 binding site, which is 100% conserved throughout vertebrate evolution, while the TEAD BS-6 binding site shows some sequence variation even among mammals. Our ChIP analyses are limited by the specificity of the antibodies used in the assay, and thus while we can show that one of the members binding *in vivo* to the enhancer is TEAD2, we cannot at this point distinguish between TEAD1 and TEAD3, one of which is also binding to the enhancer as the TEAD3 antibody, which we show recognises both TEAD1 and TEAD3, also immunoprecipitated the ECR111 sequence. Finally, we cannot be certain of the binding of TEAD4, as the antibody used also recognises TEAD1 and TEAD3, and the more specific anti-TEAD1 and anti-TEAD4 antibodies fail to generate consistent data on ChIP analyses.

Zebrafish contain at least three TEAD homologues, TEAD1 and two TEAD3s, TEAD3a and TEAD3b (Mann et al., 2007). Interestingly, in the zebrafish embryo TEAD3a is initially expressed in adaxial cells but as the differentiation of fast muscle starts at somite stage 18, TEAD3a expression is detected throughout the somite, particularly strong at the posterior ventral somitic domain, and by the end of somitogenesis is restricted to the somite borders. Although some of the antibodies that gave a positive immunohistochemistry signal in our whole-mount experiments recognise more than one member of the TEAD family in Western blots, it is interesting to note the overlap in the location of the TEAD proteins, mainly to myotome and dermomyotomal edges at 10.0 d*pc*, although at later stages, unlike the zebrafish TEAD3a, mouse TEAD proteins are detected throughout the myotome.

### Heterogeneity of signals in the dermomyotomal lips

It has been shown that the formation of the myotome in vertebrates is an ordered process in which a first wave of pioneer cells colonise the myotome from the dermomyotomal dorsomedial lip (DML) (Cinnamon et al., 1999; Denetclaw et al., 2001; Gros et al., 2004; Kahane et al., 1998; Ordahl et al., 2001; Venters and Ordahl, 2002), and the expansion of the myotome at early stages is driven only by myocytes delaminating from this source. Further growth of the myotome is accomplished by the addition of myocytes from the four lips of the dermomyotome so that the final myotome is compartmentalised in five regions depending on the source of progenitor cells (Gros et al., 2004). Our data show that this subdivision probably precedes myotome formation, as the dermomyotomal lips are already subdivided before colonisation of the myotome. In agreement with this, we have previously shown that expression of *Myf5* in the DML is driven by the EEE enhancer element (Teboul et al., 2002), while precursors in the ventro-caudal and ventro-rostral lips are subject to different environmental signals acting through ECR111. In addition, we show that the expression of *Myf5* in the somitic bud is driven by a yet uncharacterised enhancer in the − 140 kb to − 88 kb interval (Carvajal et al., 2001), which is distinct from ECR111 (this work). Enhancer elements driving expression of *Myf5* in the dorso-caudal and dorso-rostral dermomyotomal lips have not been identified at this stage, although constructs #1 and #2 from Summerbell et al. (2000) show clear *Myf5* expression in the dorso-caudal somitic domain, indicating that this uncharacterised enhancer is located close to the coding sequence of *Myf5*.

### The function of TEAD proteins in early myogenesis

TEAD proteins have been shown to be direct targets of the Hippo signalling pathway mediating YAP-activity through direct binding to this co-activator and modulating YAP-induced epithelial to mesenchymal transitions (Zhao et al., 2008) and progenitor cell proliferation and survival (Cao et al., 2008; Lian et al., 2010; Ota and Sasaki, 2008; Sawada et al., 2008). This latter function is a highly conserved pathway as overexpression of *Yorkie* (the YAP homolog) in *Drosophila*, which is also downstream of the Hippo pathway, leads to increased cell proliferation and survival (Huang et al., 2005; Zhang et al., 2008). Furthermore, TEAD proteins have also been implicated in cell growth and epithelial to mesenchymal transition through the interaction with the TAZ co-activator (Zhang et al., 2009) and in the development of neural crest derivatives through the direct activation of Pax3 (Milewski et al., 2004).

Very recently it has been shown that in the C2C12 skeletal muscle cell line activated YAP, which is unable to exit the nucleus, prevents myotube differentiation (Watt et al., 2010). Furthermore, the authors have also shown that activated YAP downregulates *MyoD* and *Mef2*, which are essential for cell cycle exit and differentiation, and induces *Myf5* expression, which has been shown to promote myoblast proliferation (Ishibashi et al., 2005). Therefore YAP function in C2C12, as in the neural tube, promotes cell proliferation and maintenance of the undifferentiated phenotype. It is therefore possible that the function of the TEAD family in the caudal and rostral lips of the dermomyotome through the ECR111 enhancer is to activate *Myf5* expression in order to allow for the proliferation of second wave progenitor myocytes, promote the growth of the ventral myotome and contribute to hypaxial muscle formation.

The following are the supplementary materials related to this article.

**Figure:**
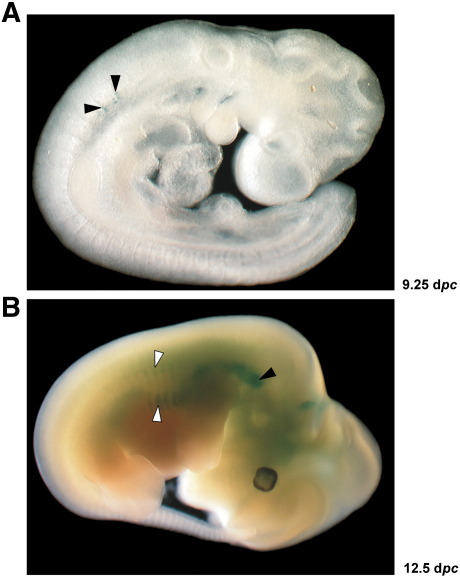
Supplementary Fig. 1.

**Figure:**
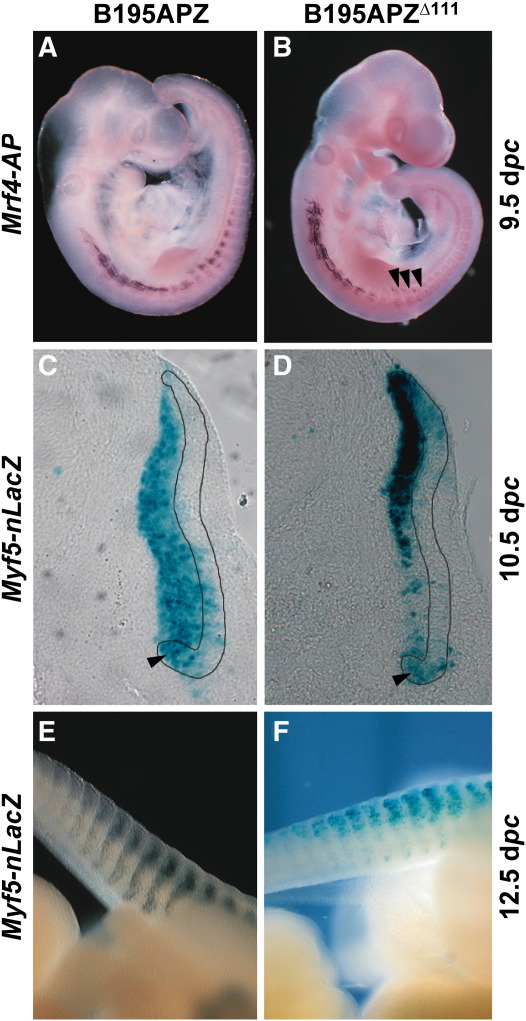
Supplementary Fig. 2.

**Figure:**
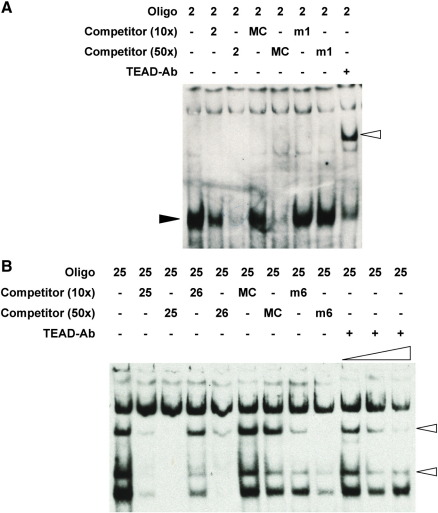
Supplementary Fig. 3.

**Figure:**
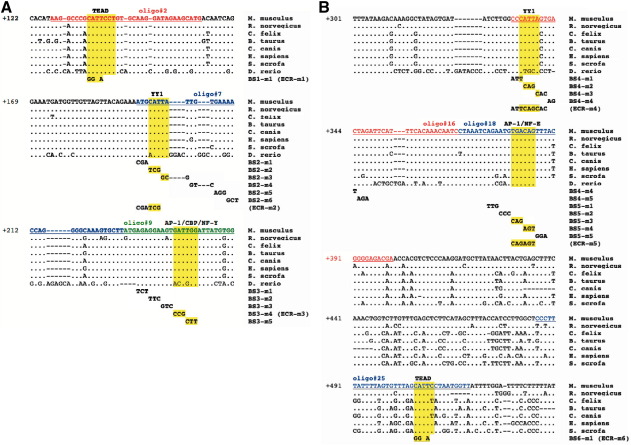
Supplementary Fig. 4.

**Figure:**
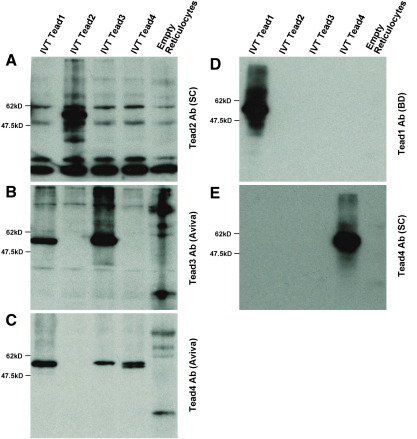
Supplementary Fig. 5.
